# 2D perfusion angiography: an alternative method to evaluate endovascular intervention for acute lower limb ischemia

**DOI:** 10.1186/s12872-022-02979-x

**Published:** 2022-12-03

**Authors:** Wanghai Li, Huimin You, Yan Zhang, Hong Zhang, Chengzhi Li

**Affiliations:** 1 Department of Interventional Radiology and Vascular Surgery, The First Affiliated Hospital of Jinan University, No. 613 West Huangpu Avenue, Guangzhou, 510630 China; 2 Department of Endocrinology, The Fifth Affiliated Hospital of Guangzhou Medical University, Guangzhou, 510700 China

**Keywords:** 2D perfusion angiography, Syngo iFlow, peripheral arterial disease, endovascular intervention

## Abstract

**Background:**

Despite advances in endovascular techniques to treat acute limb ischemia (ALI), evaluation of clinical outcomes for revascularization remains challenging, especially the accurate quantification of post-endovascular limb perfusion. This study aimed to investigate the accuracy and value of 2D perfusion angiography to evaluate endovascular intervention for ALI.

**Methods:**

A total of 47 patients with ALI were retrospectively analyzed. The transcutaneous oxygen partial pressure (TcPO2) was obtained using laser Doppler blood perfusion monitoring. The ankle-brachial index (ABI) and angiographic images were obtained before and after endovascular intervention. iFlow imaging was used to obtain color-coded images. Regions of interest (ROIs) at the femoral head, knee joint, and ankle joint were selected to obtain the time to peak (TTP). The differences in the TTP between the knee and femoral head regions (TTP difference in the knee area) and between the ankle and knee regions (TTP difference in the ankle area) were observed. The TTP, ABI, and TcPO2 between the complete response (CR), partial response (PR), no response (NR), and amputation (AM) groups were compared. The correlation between TTP changes in the ankle area (ΔTTP) and changes in ABI (ΔABI)/changes in TcPO2 (ΔTcPO2) was analyzed.

**Results:**

There was a significant increase in both TcPO2 and ABI compared with the pre-intervention values (27.75 ± 5.32 vs 40.92 ± 4.62, and 0.35 ± 0.16 vs 0.79 ± 0.15, respectively, all *p* < 0.01). The post-intervention TTP differences in the knee areas (5.12 ± 2.45 s) and ankle areas (6.93 ± 4.37 s) were significantly faster than pre-intervention TTP differences (7.03 ± 2.57 s and 10.66 ± 4.07 s, respectively, all *p* < 0.05). The post-operative TTP in the ankle area, post-operative TTP difference in the ankle area, and ΔTTP in the AM group were higher than the values in the CR and PR groups. The ΔTTP demonstrated strong correlation with ΔABI (r = −0.722, *p* < 0.01) and ΔTcPO2 (r = −0.734, *p* < 0.01).

**Conclusions:**

2D perfusion angiography with enhanced visual and quantitative analysis exhibits great potential to evaluate the efficacy of endovascular intervention, and provides a quantitative and sensitive tool to evaluate post-endovascular limb perfusion for ALI patients.

## Background

It is estimated that peripheral arterial disease (PAD) affects about 12% of the global population. With the dramatic lifestyle changes in modern society, diabetes mellitus, hypertension, hyperlipidemia, and metabolic diseases have emerged as high-risk factors for atherosclerosis, which leads to a consequent rise in the incidence of PAD [1]. Acute limb ischemia (ALI) is a common PAD, which refers to a clinical emergency with a sudden decrease in arterial perfusion in the limb due to various causes. ALI is commonly attributed to acute occlusion of the distal artery caused by embolism or hypercoagulation states, which threatens limb viability within a short interval and leaves insufficient time for new blood vessels to compensate for limb perfusion, resulting in severe ischemia of the limb, usually within 2 weeks [2].

Therefore, urgent recognition of ALI as a major vascular emergency and prompt revascularization are crucial for successful treatment. Despite advancements in endovascular techniques and vascular imaging, evaluation of clinical outcomes for revascularization remains challenging, especially accurate quantification of post-endovascular limb perfusion [3]. A thorough assessment of limb perfusion can enable timely diagnosis and evaluation of outcomes of revascularization. Digital subtraction angiography (DSA) is an integral and standard diagnostic tool to assess hemodynamic changes during endovascular intervention. However, angiography can only be used to evaluate therapeutic effects based on vascular morphology and does not truly represent microcirculatory perfusion of ischemic limb tissues. Angiography also requires a simple subjective visual interpretation, which leads to operator-dependent evaluation of endovascular intervention and significant intra/inter-observer variation [4, 5]. Thus, the lack of volumetric characterization and quantitative accuracy of angiography presents challenges in determining success of revascularization.

Two-dimensional perfusion (2DP) imaging is a new technique developed to address the deficits of traditional angiography. The Syngo iFlow imaging technique is a DSA functional imaging technique developed by Siemens Healthineers. This technique integrates the DSA sequence into an image and performs color coding, which provides an objective and quantifiable metric correlated with tissue perfusion to evaluate clinical outcomes of endovascular intervention. iFlow imaging allows clinicians to analyze and calculate a series of DSA original image data and uses time as the main parameter to display the superimposed color-coded map on an image. The time to peak (TTP) for the contrast agent in each pixel is determined based on color changes during iFlow imaging analysis. As a result, when a blood vessel is occluded, iFlow imaging shows that the peak time of the contrast agent in the blood supply area is prolonged, and the displayed color tone is colder [6, 7]. The iFlow image technique was first applied to evaluate cerebral blood perfusion [8] and later to quantitatively evaluate blood perfusion in lower limbs with chronic ischemia [9]. However, there are few studies on the application of iFlow imaging in the diagnosis and assessment of ALI and its treatment. Therefore, this study aimed to investigate the value of iFlow imaging to evaluate endovascular intervention for ALI.

## Methods

### Patient selection

**Fig. 1:**
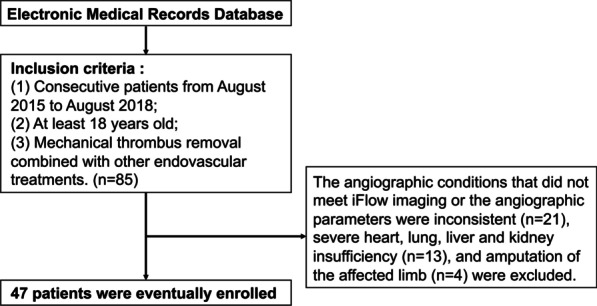
The flow diagram of the patient selection

Patients diagnosed with acute ischemia of the lower extremity and undergoing mechanical thrombectomy in our center between August 2015 and August 2018 were screened. The diagnostic criteria for ALI included acute lower extremity ischemia, a disease duration of less than 2 weeks, and obvious signs and symptoms of lower extremity ischemia (lower extremity pain, numbness, skin pallor, decreased skin temperature, and sensory disturbance). Patients who met the imaging conditions of iFlow imaging technology and who had consistent imaging parameters were further enrolled in this study. Patients with severe cardiac, pulmonary, hepatic and renal insufficiency were excluded. Patients whose affected limb has been treated with amputation were also excluded from this study. Finally, a total of 47 patients diagnosed with ALI between August 2015 and August 2018 were screened for the evaluation of endovascular intervention in this single-center study. The process for cohort establishment is shown in Fig. 1. The severity of ALI in the 47 limbs was characterized using the Rutherford classification system [10]. Demographic and clinical data were obtained from the hospital’s medical database. This retrospective study was approved by the institutional review board of the First Affiliated Hospital of Jinan University. All procedures were in accordance with the ethical standards of the national research committee and with the 1964 Helsinki Declaration.

### Endovascular intervention

Under local anesthesia, contralateral common femoral artery access was obtained with a 6-F crossover sheath. The lesion was crossed intra-luminally with a guide wire (Terumo, Japan). Next, a rheolytic percutaneous mechanical thrombectomy (PMT) device (AngioJet, Boston Scientific) was placed into the lesion. After thrombectomy, if the stenosis caused by thrombus was greater than 50% and the patient had no contraindications to catheter-directed thrombolysis (CDT), a 5-F thrombolysis catheter (Uni-Fuse, Angio Dynamics, NY) was inserted into the residual thrombus. The patient was administered urokinase (Abbokinase) via the thrombolytic catheter (400,000–800,000 U/24 h) for thrombolysis. The duration of thrombolysis was not more than 7 days, and angiography was performed every 2 to 3 days. After thrombolysis, if the stenosis was still greater than 50%, balloon angioplasty was performed. After angioplasty, stenting was implemented if the residual stenosis exceeded 50%.

### Technical success and clinical outcomes

The criteria for technical success included residual stenosis ≤ 30% and at least one artery below the knee reached the foot, as revealed by angiography. All 47 patients met these criteria for technical success in this study. The clinical outcomes included complete response (CR), partial response (PR), no response (NR), and amputation (AM). The definitions of the different outcomes were as follows: 1) CR: return to normal pulse in the distal limb after treatment, no gangrene, no sensory and motor impairment; 2) PR: recovery of distal limb pulse after treatment, weaker than the opposite side, and improvement of symptoms; 3) NR: partial recovery of blood flow in the distal artery of the limb after treatment, still with ischemic symptoms; and 4) AM: the distal end of the limb was removed and divided into major and minor amputations.

### Image acquisition

DSA images were obtained using an angiographic system (Artis Zeego; Siemens, Germany). Medical restraint bands were used to limit the movement of the affected limb during the angiography, so as not to affect the quality of image acquisition. A 5-F catheter was placed through the 6F contralateral sheath in the affected limb through the common femoral artery for all arteriographic procedures. Four successive DSA scans were performed over the entire lower extremity. The first segment contained the catheter tip and the femoral head, and the C-arm was tilted 30° medially to fully view the superficial and deep femoral arteries. The second segment observed from the anteroposterior view included the knee joint as the lower boundary of the field of view (FOV). The upper boundary of the third segment observed from the anteroposterior view was 5 cm above the upper edge of the patella. The fourth segment of the lower boundary observed from lateral view contained the entire affected foot. A power-injector was set to deliver the contrast material (320 mg iodine/ml) at a flow rate of 3 cc/s. The dose of contrast material was 9 cc for the first three segments and 15 cc for the last segment. A high-pressure syringe was set to a pressure of 300 PSI and the number of angiographic frames was 15 per second.

### DSA image postprocessing

**Fig. 2:**
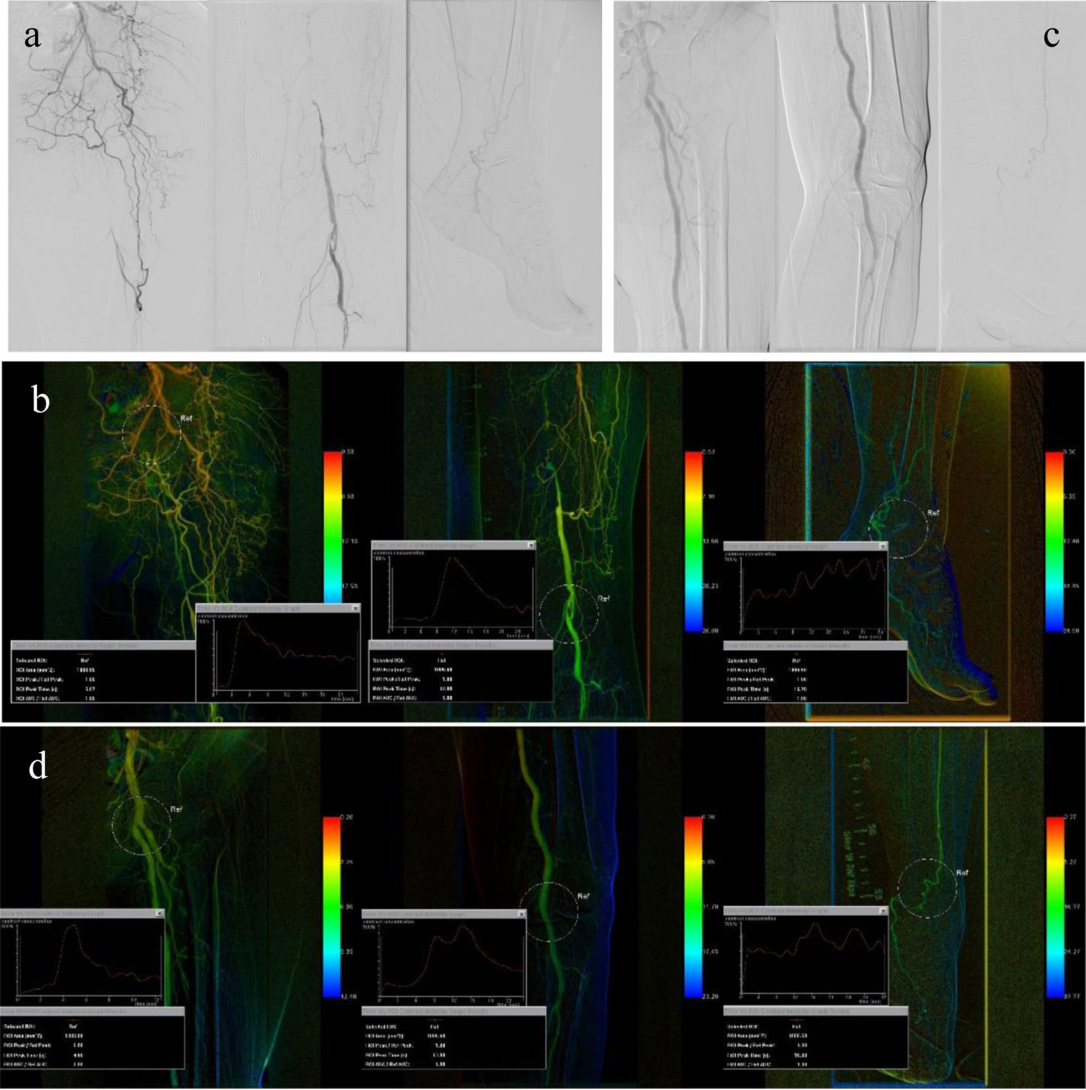
Standard DSA and iFlow images of the lower extremity. **a**, **c** Arteriography before (**a**) and after (**c**) endovascular intervention. **b**, **d** iFlow processed the same DSA sequence before (**b**) and after (**d**) endovascular intervention

The DSA data were immediately transferred to and reconstructed with a Siemens workstation (Artis Zeego Leonardo; Siemens) to generate color-coded images. Regions of interest (ROIs) in the femoral head, knee joint, and ankle joint with a size of 1000 mm^2^ were selected in the iFlow images. The TTP in each region was measured. The differences in TTP between the knee region and the femoral head region (TTP difference in the knee area) and between the ankle area and the knee area (TTP difference in the ankle area) were calculated. The above angiographic procedures and measurements were performed by two experienced interventional vascular surgeons (Fig. 2).

### Measurement of ABI and TcPO2

Pre- and post-operative ABI measurements were performed using an ABI meter. Before measurement, the patient was allowed to rest for 5–10 minutes in a supine position, and blood pressure cuffs were placed on the patient's upper arms and lower extremities at the foot and ankle, and measured using ultrasonic Doppler, with the auscultation site in the upper arm located at the pulsating brachial artery in the elbow fossa and the auscultation site in the foot and ankle located at the dorsalis pedis or posterior tibial artery. Theystolic pressure of the upper extremity was taken as the higher limit, and the ratio of the systolic pressure of the lower extremity to the systolic pressure of the upper extremity was calculated.

TcPO2 was measured using a TcPO2 detector. The patient was allowed to rest for 5–10 minutes in a supine position at room temperature before measurement, with the dorsum of the affected foot as the measurement site, avoiding skin ulcers, large blood vessels, hair, and bony prominence. The skin was cleaned at the measurement area with 75% ethanol, and after drying, take the electrode fixation patch was applied to the skin between the first toes. We then added an appropriate amount of electrode solution in the patch hole, aligned and connected the arrow mark on the electrode with the patch hole, rotated the electrode 90° clockwise, fixed it in the patch hole, waited for approximately 10 minutes for the value to stabilize, and then recorded the result.

### Statistical analysis

Continuous data are presented as the mean ± standard deviation (median), and categorical data are presented as the count (percentage). Statistical analysis was performed using SPSS 16.0 statistical software (version 16.0; IBM Corporation, USA). Any change in the TTP, TTP difference, ABI, and TcPO2 for each ROI before and after an intervention was analyzed using the paired t test. The TTP, ABI, and TcPO2 between the CR, PR, NR, and AM groups were compared using one-way analysis of variance. The correlations between ΔTTP and ΔABI/ΔTcPO2 were analyzed using Pearson’s analysis. A confidence level of 95% was used, and a *p* value < 0.05 was considered statistically significant.

## Results

**Fig. 3:**
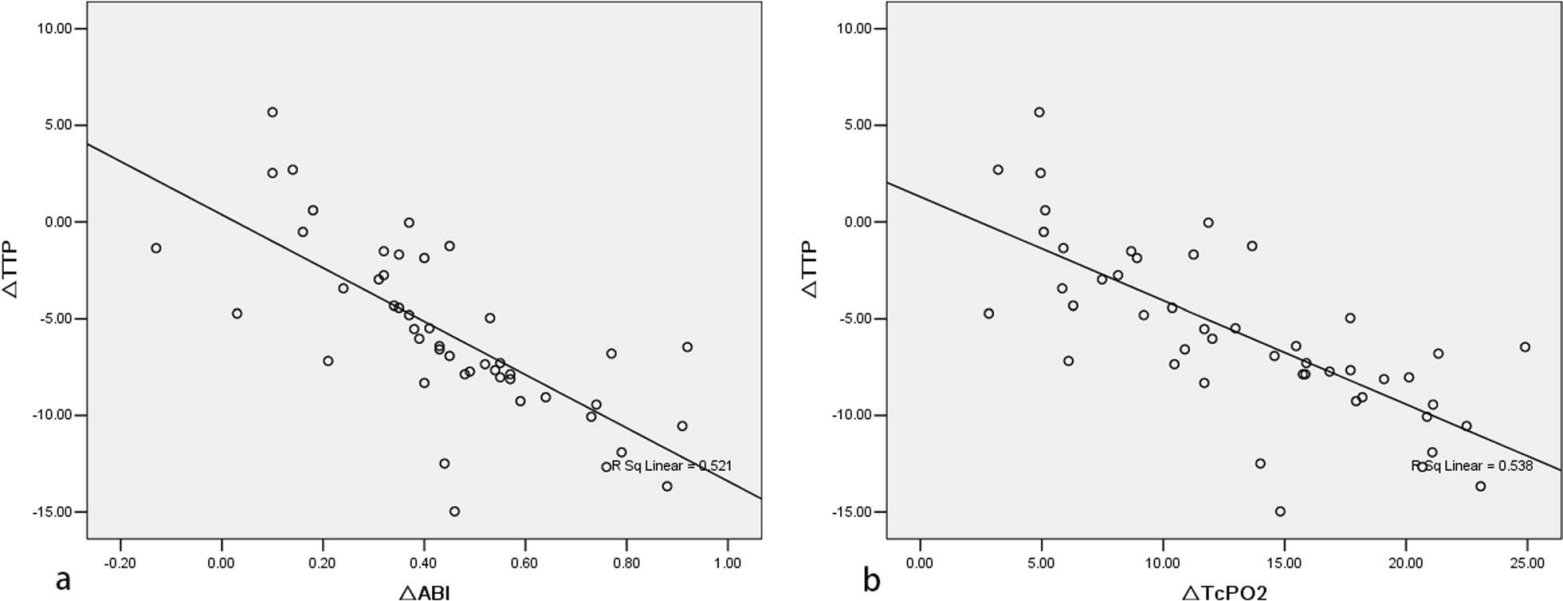
Relationship between TTP and ABI/TcPO2. **a**, **b** Pearson correlation analysis showed the correlation between the changes in TTP (ΔTTP) and ABI (ΔABI) (r = −0.722, *p* < 0.01), and the correlation between the changes in TTP (ΔTTP) and TcPO2 (ΔTcPO2) in the ankle joint area (r = −0.734, *p* < 0.01)

The baseline demographics and characteristics of the patient population are shown in Table 1. A total of 47 patients with 47 ALI limbs and a mean age of 67.53 ± 8.09 years (age range: 56 to 83 years) were registered in this study. The onset time of symptoms ranged from 3 hours to 120 hours (mean, 38.98 ± 33.58 hours). The obstruction was located in the common iliac artery in six patients, in the external iliac artery in three patients, in the common femoral artery in three patients, and distal from the common femoral artery in the remaining 30 patients. Among the 47 patients, there were 32 cases (68.09%) caused by embolization, 11 cases (23.40%) caused by thrombosis, and 4 cases (8.51%) of unknown cause. CDT was performed for 29 patients. Thirty-two patients underwent balloon dilatation. Eighteen patients eventually underwent stent implantation (the stent was placed in the iliac artery in seven cases, in the common femoral artery in two cases, in the superficial femoral artery in 11 cases, and in the popliteal artery in four cases). The technical success rate was 100%, indicating all patients recovered at least one tibial vessel with flow into the foot. Five patients (10.6%) eventually underwent amputation. Among these five patients, three had preoperative grade IIB ALI, and two had preoperative grade III ALI. Among the three patients with grade IIB ALI, one showed skin necrosis of the calf and foot after the operation. After debridement and other treatments, the wounds healed poorly, and muscle necrosis occurred. The other two patients received treatments with PMT and CDT. However, the residual thrombus remained in the inferior genicular artery, and the patients developed foot and toe ulcers and showed difficulty healing after active debridement and vacuum aspiration. These three patients eventually underwent below-the-knee amputation. The two patients with grade III ALI developed heart and kidney dysfunction after the operation, and the blood flow in the limb was poor, motor function was lost, and the patients underwent mid-thigh amputation. In this study, 13 cases (27.7%) achieved CR, and 18 cases (38.3%) achieved a PR. However, 11 cases (23.4%) were NR, and 5 cases (10.6%) underwent AM. The pre- and post-operative parameters for the 47 patients are shown in Table 2. The respective TTP differences in the knee and ankle area were 7.03 ± 2.57 s and 10.66 ± 4.07 s preoperatively and 5.12 ± 2.45 s and 6.93 ± 4.37 s postoperatively (all *p* < 0.05). The post-operative TTP in the ankle area, post-operative TTP difference in the ankle area, and ΔTTP in the AM group are higher than the values in the CR and PR groups (Table 3). Pearson correlation analysis showed that ΔTTP was negatively correlated with ΔABI and ΔTcPO2 (Fig. 3).

**Table 1:** Demographic and clinical characteristics of 47 patients ALI

Mean age (y)	67.53 ± 8.09
Male (%)	32 (68.1)
Onset time (h)	38.98 ± 33.58
Rutherford classification	
Category IIA	8 (17.0)
Category IIB	28 (59.6)
Category III	11 (23.4)
Hypertension	30 (63.8)
Diabetes mellitus	14 (29.8)
Coronary artery disease	20 (42.6)
Atrial fibrillation	25 (53.2)
Level of obstruction	
Common iliac artery	6 (12.8)
External iliac artery	3 (6.4)
Common femoral artery	8 (17.0)
Superficial femoral artery	14 (29.8)
Popliteal artery	16 (34.0)
CDT	29 (61.7)
Balloon dilatation	32 (68.1)
Adjuvant stenting	18 (38.3)

*CDT* catheter-directed thrombolysis

**Table 2:** Comparison of pre- and post-operative parameters of patients

Measurement	Pre-operative	Pre-operative	*t*	*p*
Femoral head TTP (s)	4.46 ± 1.05	4.71 ± 1.04	−1.715	> 0.05
Knee joint TTP (s)	11.49 ± 2.27	9.82 ± 2.46	7.670	< 0.01
Ankle joint TTP (s)	22.15 ± 2.98	16.76 ± 3.93	8.980	< 0.01
TTP difference in knee area (s)	7.03 ± 2.57	5.12 ± 2.45	7.479	< 0.01
TTP difference in ankle area (s)	10.66 ± 4.07	6.93 ± 4.37	3.809	< 0.01
TcPO2 (mmHg)	27.75 ± 5.32	40.92 ± 4.62	−14.994	< 0.01
ABI (%)	0.35 ± 0.16	0.79 ± 0.15	−13.238	< 0.01

TTP = time to peak

TcPO2 = transcutaneous oxygen partial pressure

ABI = ankle-brachial index

**Table 3:** Comparison of patients with different clinical outcomes

	CR	PR	NR	AM	*p* value
Post-operative TTP in the ankle area	14.94 ± 2.93	16.01 ± 4.26	18.53 ± 3.25	21.74 ± 2.53	0.003
Post-operative TTP difference in the ankle area	4.11 ± 3.45	7.76 ± 3.14	10.42 ± 3.91	12.83 ± 1.79	0.000
Post-operative ABI	0.82 ± 0.12	0.85 ± 0.14	0.75 ± 0.15	0.62 ± 0.08	0.007
Post-operative TcPO2	42.22 ± 2.30	42.38 ± 4.13	39.37 ± 6.32	35.70 ± 1.09	0.011
ΔTTP	−7.49 ± 3.31	−7.14 ± 4.33	−2.84 ± 4.58	−2.75 ± 2.14	0.008
ΔABI	0.61 ± 0.23	0.39 ± 0.21	0.40 ± 0.23	0.34 ± 0.16	0.025
ΔTcPO2	16.15 ± 5.21	13.96 ± 6.45	10.73 ± 4.84	7.94 ± 4.23	0.023

CR: complete response; PR: partial response; NR: no response; AM: amputation.

TTP = time to peak

TcPO2 = transcutaneous oxygen partial pressure

ABI = ankle-brachial index

## Discussion

Our study showed that 2D perfusion angiography enables quantitative analysis of changes in tissue perfusion. The limb perfusion across all patients in this study was reflected accurately by iFlow imaging during endovascular intervention for ALI. A significant increase in TTP differences in both the knee and ankle areas was observed after endovascular intervention, and the ΔTTP showed strong correlations with ΔABI and ΔTcPO2. The post-operative TTP in the ankle area was significantly higher in patients in the AM group compared to patients in the CR and PR groups. Our data demonstrate that 2D perfusion angiography provides valuable insights for evaluating perfusion states in patients with ALI, especially in real-time evaluation of endovascular intervention.

ALI is a clinical emergency that requires urgent diagnosis and effective treatment. With the development of interventional instruments and techniques, endovascular intervention has become an important therapy for ALI and is increasingly used in the clinical rescue of limbs to avoid amputation [11]. Conventional angiography establishes an image to delineate the intraluminal flow status, which enables the diagnosis of disease severity and assessment of endovascular intervention. However, this modality allows only visualization and qualitative assessment of the blood flow status of blood vessels, and lacks quantitative ability to reflect the microcirculation and tissue perfusion [12]. iFlow imaging emerged as an innovated technique that provides the visualization of anatomy as well as functional information of the microcirculation and tissue perfusion without increasing the amount of contrast agent and radiation dose in patients.

iFlow is DSA functional imaging software that can be used to calculate and process DSA contrast images. iFlow detects the signal intensity of contrast material flowing through a specified ROI and generates a wealth of quantitative information, including the area under the curve (AUC), arrival time (AT), leg transit time (LTT), mean transit time (MTT), TTP, and wash-in rate (WIR), which allows accurate assessment of tissue perfusion [13]. Hence, iFlow images contain information representing the perfusion of the tissue, and thus point and regional measurements can be used to quantify the tissue perfusion parameters in ROIs [14]. Numerous reports have shown that iFlow imaging technology has been applied to evaluate the efficacy of endovascular intervention for lower extremity arterial obstructive disease, and has been confirmed to accurately reflect hemodynamic changes after angioplasty [15–17]. Jens et al. [15] used post-processing angiographic perfusion imaging to generate a time-density curve, which accurately captured changes in foot perfusion in patients with severe limb ischemia after treatment. However, the current application of iFlow technology to assess limb ischemia is limited due to chronic limb ischemia [9], and there have been no reports on the application of iFlow technology for the assessment of ALI. Current methods for assessing ALI severity are not sufficient to fully reveal the microcirculation and tissue perfusion.

It is challenging to determine vascular status due to vascular occlusion in the limb before endovascular intervention, and selecting only the muscle as the ROI does not directly reflect improvements in blood flow after endovascular intervention. Therefore, in the present study, a region containing muscles and blood vessels was selected as the ROI, and a bone marker was included to determine the position of the ROI before and after intervention. Considering the physiologic and anatomic differences across patients, such as volume status and blood pressure, which may lead to wide variations in TTP in the assigned ROI, the TTP differences in the knee area and the ankle joint area derived from TTP were used as observation indexes. The guidelines [11, 18] recommend amputation for patients with grade III ALI. However, in clinical practice, some patients often decline amputation, and thus endovascular intervention is required to save the limb. In the present study, five of the 47 patients were eventually treated with amputation. The reported amputation rate in ALI patients after endovascular intervention ranges from 10–15% [19, 20], which is consistent with our study’s amputation rate of 10.6%. In addition, we found significant differences in both TTP and TTP differences between the knee and ankle areas before and after endovascular intervention. ABI and TcPO2 before and after intervention were also significantly different. Furthermore we found significant differences in both post-operative TTP in the ankle area, post-operative TTP difference in the ankle area, and ΔTTP differences between the AM group and CR/PR groups. These findings suggest that (1) blood flow perfusion of the affected limb significantly improved after endovascular intervention, and (2) TTP determined by iFlow imaging can faithfully reflect therapeutic effects at the affected limb, which could be used to predict the prognosis of the affected limb. Intriguingly, no significant difference in the pre- and post-intervention TTP of the femoral head region was observed, which may be due to a large variation in the location of the plane of obstruction among different patients. Therefore, complete opening of the affected blood vessels of the iliac axis had no significant effect on blood flow in the common femoral artery.

The TTP of the knee and ankle of normal patients was not evaluated in this study because it would have been necessary to perform invasive angiography on normal patients to obtain the TTP. Rather, limb TTP was used as a reference because the contralateral limb itself may also have vascular disease.

Our study has some limitations. First, the study was a retrospective study with a small sample size. Further validation in prospective studies with a large cohort of patients is needed in the future to draw definitive conclusions. Second, iFlow image quality is affected by angiographic limb movement, which may have caused measurement errors. Lastly, the purpose of this study was to evaluate the accuracy and value of iFlow technology in the assessment of perioperative tissue perfusion in ALI patients, and further studies are needed to investigate whether iFlow technology can be used to predict clinical outcomes of ALI.

## Conclusions

In conclusion, 2D perfusion angiography with enhanced visual and quantitative analysis exhibits great potential for evaluating the efficacy of endovascular intervention. However, whether TTP can be used as a marker to guide endovascular intervention warrants further study.

## Data Availability

The datasets used and/or analyzed during the current study are available from the corresponding author upon reasonable request.

## Abbreviations

ALI: Acute limb ischemia
2DP: Two-dimensional perfusion
TcPO2: Transcutaneous oxygen partial pressure
ABI: Ankle-brachial index
ROIs: Regions of interest
TTP: Time to peak
PAD: Peripheral arterial disease
DSA: Digital subtraction angiography
PMT: Percutaneous mechanical thrombectomy
CDT: Catheter-directed thrombolysis
FOV: Field of view
AUC: Area under the curve
AT: Arrival time
LTT: Leg transit time
MTT: Mean transit time
WIR: Wash-in rate
ΔTTP: TTP changes in the ankle area
ΔABI: The changes in the ABI
ΔTcPO2: The changes in the TcPO2
CR: Complete response
PR: Partial response
NR: No response
AM: Amputation
